# Assessing anemia in stroke patients through virtual non-contrast imaging with photon-counting detector CT: validation on supra-aortic vessel CT-Angiography

**DOI:** 10.1007/s00234-025-03620-2

**Published:** 2025-04-24

**Authors:** Guilherme A. Quint, Josua A. Decker, Abraham Cortes, Ansgar Berlis, Christoph J. Maurer

**Affiliations:** 1 Department of Diagnostic and Interventional Neuroradiology, University Hospital Augsburg, Stenglinstraße 2, 86156 Augsburg, Germany; 2 Department of Diagnostic and Interventional Radiology, University Hospital Augsburg, Stenglinstraße 2, 86156 Augsburg, Germany

**Keywords:** Anemia detection, Photon-counting detector CT, Virtual-non-contrast-imaging, Stroke, Supra-aortic vessels

## Abstract

**Background and Purpose:**

Anemia is a common comorbidity in stroke patients, traditionally detected via blood tests. This study evaluates the feasibility of using virtual non-contrast (VNC) imaging from photon counting detector-CT (PCD-CT) angiography to detect anemia and identifies the optimal anatomical site for assessment.

**Materials and Methods:**

In this retrospective study of 80 patients undergoing PCD-CT angiography of supra-aortic vessels, VNC series were analyzed at various anatomical sites, including the jugular veins, aorta, and cerebral sinuses. Correlations between serum hemoglobin (Hb) levels and VNC Hounsfield Unit (HU) values were assessed using Pearson’s coefficients. Linear regression and ROC analysis evaluated diagnostic performance.ResultsThe jugular veins showed the strongest correlation between VNC HU values and Hb levels (R^2^ = 0.49, p < 0.001), with weaker correlations in arterial vessels like the aorta (R^2^ = 0.11, p < 0.001). ROC analysis of jugular vein VNC values yielded an AUC of 0.79 for anemia detection. Correlation strength declined with longer intervals between imaging and blood tests, suggesting temporal Hb variability.

**Conclusions:**

VNC imaging in CT angiography is a feasible method for detecting anemia, with the jugular veins providing the most reliable site for assessment. VNC imaging could be a valuable alternative when blood tests are delayed or unavailable.

**Supplementary Information:**

The online version contains supplementary material available at 10.1007/s00234-025-03620-2.

## Introduction

Anemia is commonly observed as a comorbidity in patients with acute ischemic stroke (AIS), affecting up to 40% of this population [1]. It is associated with worse outcomes, not only by increasing mortality risk but also by contributing to poorer functional recovery, potentially through mechanisms such as reduced oxygen delivery, altered blood viscosity, and impaired cerebral autoregulation [2–5]. Therefore, the recognition and management of anemia is critical to the treatment and prognosis of stroke patients [6].

A well-established linear correlation exists between serum hemoglobin levels and CT attenuation values on unenhanced chest CT scans [7–11]. More recently, contrast-enhanced spectral CT and photon-counting detector CT (PCD-CT) have demonstrated the ability to reliably predict anemia in patients undergoing chest and abdominal imaging using virtual non-contrast (VNC) series generated during post-processing [12–14]. In these VNC images, the iodine concentration in each voxel is quantified and subtracted by analyzing X-ray absorption at different energy levels, creating a virtual unenhanced dataset for subsequent measurements. However, in neurological patients, anemia assessments are typically derived from unenhanced cranial CTs [15–18].

Validation of anemia detection using VNC series outside of chest and abdominal imaging remains limited. Existing data suggest that not all anatomical regions are equally suited for evaluation, and combining measurements from multiple locations may improve the accuracy of anemia detection [13]. While anemia can be assessed using standard non-contrast CT of the head, intracranial measurements—particularly in the dural venous sinuses—may be affected by beam-hardening artifacts. These artifacts, caused by the absorption of low-energy photons as X-rays pass through dense structures like the cranial bone, can distort CT attenuation values and potentially impact reproducibility [19]. PCD-CT with VNC imaging offers the advantage of reducing the influence of beam-hardening effects by enabling attenuation measurements in regions less affected by bony structures, such as the cervical vasculature. By extending the evaluation beyond the intracranial compartment to include the neck, we can assess multiple vascular sites and determine the most reliable location for anemia detection.

Therefore, the aim of this study is twofold: first, to evaluate the correlation between CT attenuation values on VNC series derived from CT angiography using PCD-CT and serum hemoglobin levels; second, to identify the optimal anatomical site for this analysis.

## Methods

This single-center, retrospective, observational study was approved by the Institutional Review Board the Ludwig-Maximilian-University of Munich, Germany (reference: 22–0456). The study adhered to the ethical standards outlined in the Declaration of Helsinki (1964) and subsequent revisions. Due to the retrospective design and irreversible anonymization of patient data, the requirement for individual informed consent was waived by the Review Board.

### Patients

All patients who underwent arterial phase CT angiography of the supra-aortic brain-supplying vessels using PCD-CT (NAEOTOM Alpha, Siemens Healthineers, Erlangen, Germany) between July 1, 2021, and May 30, 2022, were screened. Exclusion criteria included missing or outdated (> 7 days) complete blood count, the presence of significant beam-hardening artifacts from foreign material due to prior cervical spine surgery, or incomplete coverage of the supra-aortic brain-supplying vessels.

The definition of the world health organization (WHO) for anemia was used. Values are defined as healthy (i.e. no anemia), mild, moderate, and severe anemia with hemoglobin levels of ≥ 12.0, < 12.0–11.0, < 11.0––8.0 and < 8.0 mg/dl for women and ≥ 13.0, < 13.0–11.0, < 11.0–8.0 and < 8.0 mg/dl for men [20].

### Scanning protocol

All scans were conducted on a PCD-CT as part of routine clinical practice. According to our institutional standard protocol for head and neck CTA, a monophasic contrast injection protocol was used, consisting of 60 mL of iodinated contrast agent (iopromide; Ultravist 300 mgI/mL, Bayer, Leverkusen, Germany) administered via an antecubital vein, followed by a 20 mL saline flush. Both contrast and saline were injected at a flow rate of 4.5 mL/s. The scan was triggered by the contrast bolus in the ascending aorta once an attenuation threshold of 120 HU was reached. Patients were scanned in a caudocranial direction while in the supine position, from the ascending aorta to the vertex. The scanning parameters were as follows: acquisition mode with spectral information readout (QuantumPlus, Siemens), 120 kVp tube voltage with automatic tube current modulation (Care DOSE 4D, Siemens Healthineers), 0.25 s rotation time, 0.8 pitch, and 144 × 0.4 mm collimation. VNC images were generated using a dual-energy material decomposition algorithm, which subtracts the iodine component from the image data to approximate a non-contrast scan. This process was performed using the vendor-provided reconstruction algorithm. For VNC image generation, a two-material decomposition model was applied, distinguishing between iodine and soft tissue. This method relies on the spectral separation provided by PCD-CT to selectively remove the iodine signal while preserving the underlying tissue attenuation. Full-volume spectral series were generated using a soft-tissue kernel (Qr40f, Quantum iterative reconstruction (QIR) level 3) and saved in an enhanced DICOM format containing full spectral information (SPP, spectral post-processing). The slice thickness was 0.8 mm with an increment of 1 mm.

### Image processing and analysis

Image analysis was performed using a dedicated workstation (Syngo.via VB60A, MM reading workflow; Siemens Healthineers, Erlangen, Germany) by a radiology resident with 1.5 years’ experience in computed tomography angiography of the head and neck (GAQ) blinded to both laboratory and clinical data. Regions of interest (ROIs) were delineated at selected anatomical locations to ensure reliable attenuation measurements while minimizing artifacts. To balances arterial and venous compartments, following sites were predefined: Arterial ROIs included the ascending aorta, brachiocephalic trunk, and descending aorta, as these vessels provide stable contrast attenuation and serve as major conduits for systemic circulation. The right and left jugular veins were measured bilaterally at the hyoid bone level to reduce potential artifacts from the shoulders and mandible, ensuring consistent and reproducible measurements. Intracranial venous structures were included due to their potential role in hemoglobin estimation, as they contain slow-flowing blood that may better reflect systemic hemoglobin levels. Specifically, the sigmoid sinuses, confluence of sinuses, superior sagittal sinus, and great cerebral vein were analyzed to assess attenuation in dural venous sinuses, which have been previously studied in the context of anemia evaluation. ROIs were defined to cover approximately two-thirds of the vessel diameter, minimizing the risk of partial volume effects from surrounding tissues. The average ROI sizes per location were 4.4 cm² for the ascending aorta, 3.5 cm² for the brachiocephalic trunk, 3.0 cm² for the descending aorta, 1.0 cm² for the right internal jugular vein, 1.1 cm² for the left internal jugular vein, 0.3 cm² for the sigmoid sinus, 0.4 cm² for the confluence of sinuses, 0.2 cm² for the great cerebral vein (Vein of Galen), and 0.3 cm² for the superior sagittal sinus. After placing each ROI on the SPP series, the software automatically performs ROI-based CT value analysis for both the 70 keV Virtual Monoenergetic Imaging (VMI) series (CT-values 70 keV) and the VNC series (CT-values VNC), which are computed simultaneously in the background. The 70 keV VMI series was chosen because it has been shown to most closely resemble conventional 120-kVp polychromatic images while offering improved signal-to-noise and contrast-to-noise ratios [X^21^, ^22^].

### Statistical analysis

Data were analyzed using Python version 3.11.8 utilizing the SciPy, pandas, numpy and matplotlib libraries for data manipulation, statistical computations and visualization. Pearson’s correlation coefficients were calculated between Hb levels and VNC HU values for each anatomical region, with a focus on the jugular veins, aorta, great cerebral vein and the venous sinuses. Combined VNC values from multiple key regions were also assessed for their predictive power. Simple and multiple linear regression models evaluated the relationship between VNC values and Hb, with R² values reported to determine the variance explained. Multiple linear regression was used to evaluate the association between VNC attenuation values and hemoglobin levels, adjusting for potential confounders such as age and sex. While multiple measurements per patient were included, the model did not explicitly account for intra-patient clustering. Receiver Operating Characteristic (ROC) analysis assessed the ability of jugular VNC values to distinguish anemic (Hb < 12 mg/dL for women and < 13 mg/dl for men) from non-anemic patients, with the area under the curve (AUC) used to measure diagnostic accuracy. A p-value ≤ 0.05 was considered to indicate statistically significant differences for all tests.

## Results

Out of the initial selection of 111 patients, 31 were excluded based on the predefined exclusion criteria: missing or complete blood count older than 7 days (*n* = 15), CT angiography (CTA) limited to intracranial vessels only (*n* = 1), CTA confined to extracranial areas only (*n* = 1), excessive interference due to cervical spine surgery (*n* = 3), and CTA omitting some of the points of interest (*n* = 11). 80 patients remained for the final analysis. The mean age was 70.3 years (SD ± 12.4 years), ranging from 33 to 92 years. Among these, 48 were male (mean age 69.75 years ± 13.4, ranging from 33 to 88 years) and 32 were female participants (mean age 71.4 years ± 10.7, ranging from 35 to 92 years).

**Fig. 1:**
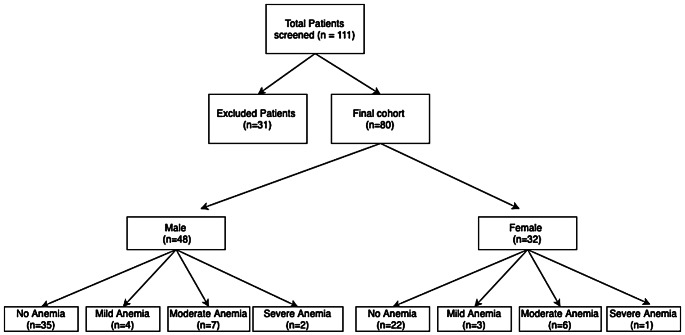
Flowchart of patient selection and distribution of anemia characteristics

Patient selection and anemia characteristics are shown in the flowchart in Fig. 1. Patient demographics and anemia classification, are shown in Table 1. The VNC and monoenergetic image VMI attenuation values are provided in the supplementary material (Supplementary Table S1).

**Table 1:** Patients’ characteristics

Characteristics	*n* = 80
**Age Mean (SD)**	70.3 (± 12.4)
**Sex**,** n (%)**	
Male	48 (60%)
Female	32 (40%)
**Anemia**,** n (%)**	
No Anemia	57 (71.25%)
Mild Anemia	7 (8.75%)
Moderate Anemia	13 (16.25%)
Severe Anemia	3 (3.75%)

Correlations between regions varied significantly. The relationships between hemoglobin levels and VNC values at the points of interest are summarized in Table 2. Notably, measurements in the jugular vein demonstrated the strongest and highly significant correlation with hemoglobin levels, exhibiting a correlation coefficient of 0.70 and a coefficient of determination (R²) of 0.49. Linear regression analysis confirmed these findings, revealing a strong association between CT attenuation values and hemoglobin levels in the jugular vein, with a regression coefficient of 2.05 HU/mg/dl (*p* < 0.001) and an R² of 0.49. The ROC analysis demonstrated an AUC of 0.88 (Fig. 1). Complete results of the linear regression analysis are provided as electronic supplementary material (Supplementary Table S2).

**Table 2:** Pearson correlation coefficient and R² value depending on location

VNC Region	Correlation (*r*)	*R*² Value	*p*-value
Jugular vein	0.70	0.49	< 0.001
Great cerebral vein	0.48	0.23	< 0.001
Sigmoid sinus	0.41	0.17	< 0.001
Confluens sinuum	0.39	0.15	< 0.001
Superior sagittal sinus	0.33	0.11	0.003
Descending aorta	0.28	0.08	0.012
Asecending aorta	0.27	0.07	0.014
Brachiocepahlic trunc	0.26	0.07	0.018

**Fig. 2:**
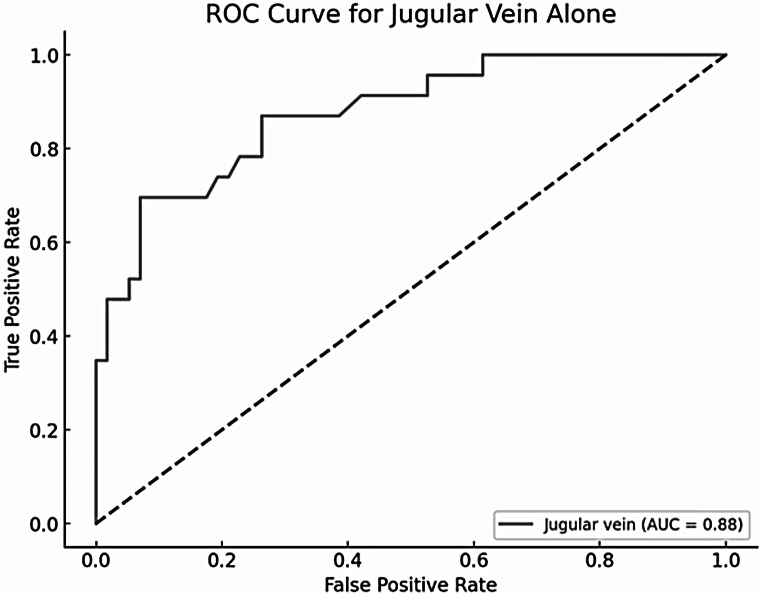
Diagnostic performance of VNC measurement in the jugular vein for predicting anemia (ROC: Receiver Operating Characteristic, AUC: Area under the curve

We found a substantial difference in the VNC values of the jugular vein between patients without anemia and those with mild, moderate, and severe anemia. Figure 2 illustrates the distribution of VNC attenuation values in the jugular vein, categorized by anemia classification. Patients with anemia exhibit lower VNC attenuation values compared to non-anemic patients, as indicated by the median and interquartile range.

**Fig. 3:**
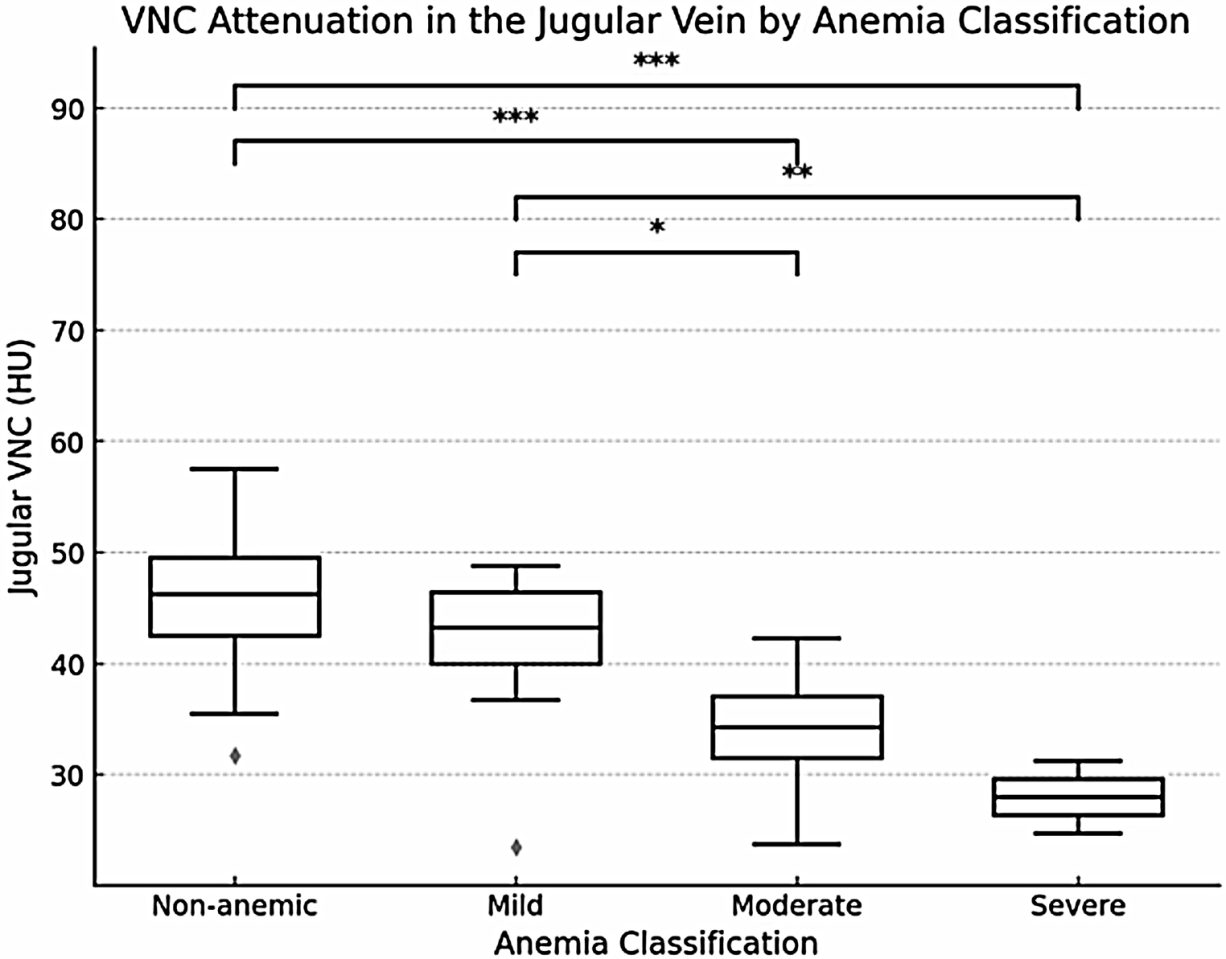
Boxplot of VNC Attenuation in the jugular vein and Anemia classification; *p < 0.05, **p < 0.01, ***p < 0.001 for pairwise comparison (Tukey’s pos hoc test)

**Fig. 4:**
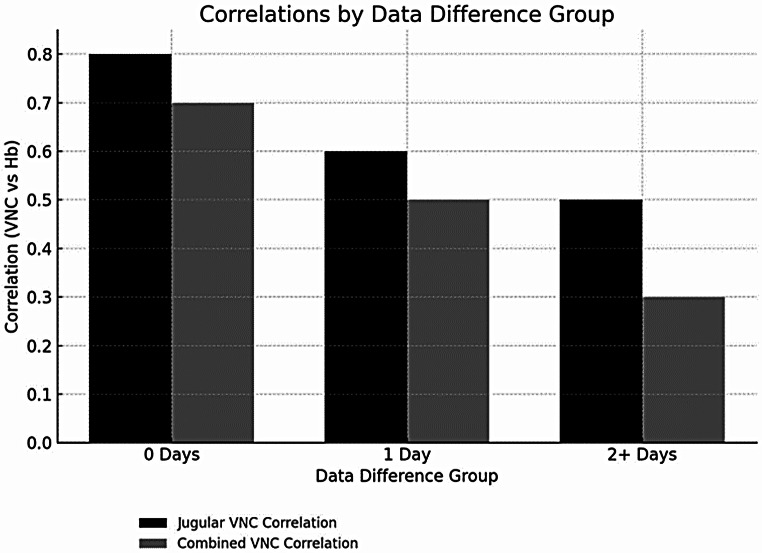
Time dependency of correlation for jugular vein

We also found varying degrees of correlation between VNC values and hemoglobin levels across different time intervals. The greater the time difference between CT scan and blood withdrawal the weaker was the correlation, suggesting that the temporal aspect plays a crucial role in the accuracy of anemia detection using VNC values (Fig. 3). To estimate the potential bias introduced by temporal variability, we performed a subgroup analysis including only patients where hemoglobin values were obtained within 24 h of the CT scan (day 0 group, *n* = 39). In this subgroup, the correlation between jugular vein VNC values and hemoglobin was notably stronger (*r* = 0.80, R² = 0.64, *p* < 0.001) compared to the full cohort (*r* = 0.70, R² = 0.49). These findings suggest that the time delay between imaging and blood sampling significantly affects the correlation strength, emphasizing the importance of minimizing this interval for accurate anemia estimation using VNC measurements (See Fig. 4).

The area under the ROC curve for predicting severe anemia (< 8 mg/dl) for the complete cohort using VNC values of the jugular vein was calculated to be approximately 0.94. An optimal cut-off value of 36.3 HU was identified, providing the best balance between sensitivity and specificity. At this threshold, the sensitivity was 100%, correctly identifying all patients with severe anemia, and the specificity was approximately 94%, correctly classifying the majority of patients without severe anemia.

## Discussion

Our data demonstrate that anemia detection using VNC series on CT angiography of the supra-aortic vessels is feasible, with the best results obtained from measurements in the jugular vein. However, the correlation decreased significantly in other locations, particularly in arterial vessels such as the aorta and brachiocephalic artery. This discrepancy between venous and arterial vessels in VNC series has been previously described [23–25]. The significantly higher attenuation in arterial-phase CTA compared to true non-contrast imaging may reduce accuracy and hinder anemia detection. In contrast, venous structures such as the jugular vein and cerebral sinuses are often only partially opacified during arterial CTA, for example due to the duration of the contrast bolus, differences in cardiac output, and inter-patient variability. Although TNC imaging remains the gold standard for measuring baseline attenuation, previous studies have demonstrated that VNC imaging can approximate TNC values with high accuracy while avoiding additional radiation exposure [26–28]. A direct comparison between TNC and VNC images was not included in this study, since a full dataset of TNC images is not available. Nonetheless, a direct comparison between VNC and TNC scans in the context of anemia detection would provide valuable confirmation of our findings and should be considered in future studies.

Measurements in the jugular vein provided a robust evaluation of hemoglobin levels in our study and effectively distinguished between patients with and without severe anemia. However, the extent of the correlation appears to vary between studies. Decker et al. reported a correlation of R² = 0.80, whereas the data from Zopfs et al. showed a correlation more in line with our findings (R² = 0.54) [12, 13]. Since we used the same system as Decker et al., differences in CT system technology, detector configuration, or post-processing are unlikely to explain this discrepancy. The more heterogeneous data and the inclusion of CT angiographies in the study by Zopfs et al. may have contributed to the lower correlation. Furthermore, patient-specific factors may contribute to these differences. For instance, Fallah Arzpeyma et al. analyzed 1,680 head CTs and found that hematocrit, serum hemoglobin, and blood urea nitrogen levels all contributed significantly to the attenuation of cerebral venous sinuses. However, our results are not directly comparable, as they used unenhanced head CTs and measured CT values in large cerebral sinuses [18]. Our data suggest that measurements in the jugular vein may be more reliable than those in the cerebral sinuses. Potential limitations of measuring in large sinuses include smaller ROIs and proximity to cranial bones, which may introduce noise and artifacts, impacting accuracy. So, measurements in the jugular veins, where a large ROI can be placed without skull bone artefacts and with venous or nearly unenhanced HU values, may be a promising site for the assessment of anemia. Our findings suggest that venous attenuation in VNC imaging correlates with anemia, raising the question of whether similar patterns could be observed using standard TNC imaging. TNC imaging remains the reference standard for non-contrast attenuation measurements. However, PCD-CT has been shown to produce reliable VNC images with superior quality compared to VNC reconstructions from conventional dual-energy CT with integrating detectors [29, 30]. As our study is based on contrast-enhanced CT angiography datasets, direct comparisons with TNC imaging were not feasible. Further studies comparing these modalities head-to-head are warranted. Additionally, exploring anatomical factors that influence measurement accuracy, such as the optimal site for VNC measurements, remains also a critical area for further investigation. The different detector technologies between conventional CT and PCD-CT may also influence the results. To our knowledge, however, validation of unenhanced PCD-CT data and its correlation with anemia has yet to be conducted.

In stroke patients, the typical protocol involves both unenhanced CT and CT angiography of the supra-aortic vessels, so TNC data is generally available. However, in clinical scenarios where native scans are not available, VNC images derived from CTA datasets may offer valuable diagnostic information. Furthermore, with technological advancements in CT, it is conceivable that unenhanced head CTs could eventually be replaced by VNC series [31].

Our study also revealed temporal variability in the correlation between VNC values and serum hemoglobin levels. The correlation diminished as the time gap between imaging and blood tests increased, likely due to hemoglobin fluctuations during hospitalization in stroke patients. Possible contributors to this variability include differences in hydration status, impaired kidney function, medication effects, and infections. Our subgroup analysis of patients with co-terminous laboratory blood values (i.e., those with hemoglobin measurements within 24 h of imaging) demonstrated a stronger correlation (R² = 0.64) compared to the full cohort (R² = 0.49). This suggests that temporal variability in hemoglobin levels during hospitalization may introduce bias when estimating anemia from VNC series. Therefore, minimizing the time difference between imaging and blood tests is crucial for future research to confirm our findings.

While full blood count tests are widely available, rapid, and inexpensive, offering near-immediate hemoglobin results, the ability to suspect anemia through imaging immediately after acquisition could potentially surpass the speed of modern blood tests. Additionally, in preclinical settings such as mobile stroke units equipped with CT scanners in ambulances, the correlation between TNC or VNC values and hematocrit or anemia is particularly useful when blood tests are unavailable or time-consuming. Furthermore, it may provide a redundancy system, preventing delays in diagnosis. In cases where laboratory data is delayed or inaccessible, imaging could offer a fast alternative. HU-based anemia detection could serve as an initial indicator, later confirmed by laboratory data. Additionally, follow-up imaging of hospitalized patients may provide preliminary insights into anemia development during their hospital stay.

Our study has several limitations. It is a retrospective observational study of a relatively small number of patients scanned for various clinical indications, which introduces considerable bias. Furthermore, we allowed a time difference of up to one week between the scan and serum hemoglobin measurement, which could significantly affect our findings. Additionally, we did not compare our results with established methods, such as CT value measurements within the cerebral sinuses, limiting the broader applicability of our findings. However, in our exploratory analysis, we were the first to demonstrate the feasibility of detecting anemia using VNC series from CT angiography datasets of the supra-aortic vessels. This provides a foundation for future studies to refine and validate this approach in larger, more controlled patient populations.

## Conclusion

Our study demonstrates the feasibility of using VNC series from photon-counting detector CT angiography of the head and neck to detect anemia, particularly with measurements taken from the jugular vein. A cutoff value of 36.3 HU effectively distinguished patients with severe anemia from those without severe anemia.

## Electronic supplementary material

Below is the link to the electronic supplementary material.

Supplementary Material 1

Supplementary Material 2

## Data Availability

The datasets used and/or analyzed during the current study are available from the corresponding author on reasonable request.

## Abbreviations

AIS: Acute Ischemic Stroke
AUC: Area Under the Curve
Hb: Hemoglobin
HU: Hounsfield Unit
PCD: CT-Photon-Counting Detector Computed Tomography
ROC: Receiver Operating Characteristic
SD: Standard Deviation
TNC: True Non-Contrast
VMI: Virtual Monoenergetic Imaging
VNC: Virtual Non-Contrast
