# Supplementary material for: Assessing anemia in stroke patients through virtual non-contrast imaging with photon-counting detector CT: validation on supra-aortic vessel CT-Angiography
Source: Neuroradiology. 2025 Apr 24;67(8):2031–9. doi: 10.1007/s00234-025-03620-2 (PMC12494657; doi:10.1007/s00234-025-03620-2)
Supplement: Supplementary file 2 — Supplementary Material 2 [file 234_2025_3620_MOESM2_ESM.docx]

**Supplementary Table S2** Results of the linear regression depending on location

| **Region** | **Slope (β)** | **R²** | **p-value** |
| --- | --- | --- | --- |
| **Jugular Vein** | 0.24 | 0.49 | < 0.001 |
| **Great Cerebral Vein** | 0.28 | 0.23 | < 0.001 |
| **Sigmoid Sinus** | 0.19 | 0.17 | < 0.001 |
| **Confluens Sinuum** | 0.19 | 0.15 | < 0.001 |
| **Superior Sagittal Sinus** | 0.14 | 0.11 | 0.003 |
| **Ascending Aorta** | 0.13 | 0.08 | 0.019 |
| **Brachiocephalic Trunk** | 0.12 | 0.07 | 0028 |
